# Prognostic Significance of miR-205 in Endometrial Cancer

**DOI:** 10.1371/journal.pone.0035158

**Published:** 2012-04-13

**Authors:** Mihriban Karaayvaz, Cecilia Zhang, Sharon Liang, Kenneth R. Shroyer, Jingfang Ju

**Affiliations:** 1 Department of Pathology, Stony Brook University, Stony Brook, New York, United States of America; 2 Department of Physiology and Pharmacology, SUNY Downstate Medical Center, Brooklyn, New York, United States of America; 3 Department of Obstetrics/Gynecology, Hofstra North Shore-LIJ School of Medicine, Hofstra University, Hempstead, New York, United States of America; University of Louisville, United States of America

## Abstract

**Purpose:**

microRNAs have emerged as key regulators of gene expression, and their altered expression has been associated with tumorigenesis and tumor progression. Thus, microRNAs have potential as both cancer biomarkers and/or potential novel therapeutic targets. Although accumulating evidence suggests the role of aberrant microRNA expression in endometrial carcinogenesis, there are still limited data available about the prognostic significance of microRNAs in endometrial cancer. The goal of this study is to investigate the prognostic value of selected key microRNAs in endometrial cancer by the analysis of archival formalin-fixed paraffin-embedded tissues.

**Experimental Design:**

Total RNAs were extracted from 48 paired normal and endometrial tumor specimens using Trizol based approach. The expression of miR-26a, let-7g, miR-21, miR-181b, miR-200c, miR-192, miR-215, miR-200c, and miR-205 were quantified by real time qRT-PCR expression analysis. Targets of the differentially expressed miRNAs were quantified using immunohistochemistry. Statistical analysis was performed by GraphPad Prism 5.0.

**Results:**

The expression levels of miR-200c (P<0.0001) and miR-205 (P<0.0001) were significantly increased in endometrial tumors compared to normal tissues. Kaplan-Meier survival analysis revealed that high levels of miR-205 expression were associated with poor patient overall survival (hazard ratio, 0.377; Logrank test, P = 0.028). Furthermore, decreased expression of a miR-205 target PTEN was detected in endometrial cancer tissues compared to normal tissues.

**Conclusion:**

miR-205 holds a unique potential as a prognostic biomarker in endometrial cancer.

## Introduction

Endometrial cancer is the most common malignancy of the female genital tract in the United States [1]. The overall five-year survival of endometrial cancer is approximately 80% among all stages. Although it has a relatively low rate of mortality compared to other gynecologic cancers, certain histologic types are highly invasive, frequently metastatic and have poor survival rates. The most dominant subtype, endometrioid endometrial cancer (EEC) accounts for approximately 80% of the cases. Surgery is typically curative with the majority of these cases diagnosed at an early stage. In contrast, patients with advanced stage or nonendometrioid endometrial cancers tend to display more aggressive characteristics and have a much less favorable prognosis [2]. Although the morphological alterations reflect important insights into endometrial cancer, there is an urgent need for highly sensitive and specific molecular prognostic biomarkers to better predict the outcome of endometrial cancer, especially in more than 25% of the endometrioid endometrial tumors with high risk to develop more aggressive diseases.

Recently, molecular studies have identified microsatellite instability and mutations in PTEN, K-ras, beta-catenin, p53, HER-2/neu, p16 and E-cadherin genes in cases of endometrial cancer [3]. Particularly, the incidence of PTEN mutations has been reported to be the highest among other genetic alterations, existing in 34–55% of endometrial cancers [4]–[6]. PTEN is an important tumor suppressor in endometrial cancer, and the identification of these molecular rearrangements can aid in better understanding of endometrial cancer biology and can also lead to the discovery of novel molecular targets for the detection, prognosis, and therapy. Although these genetic alterations have been described as important, they are not universally present in all cases suggesting that epigenetic mechanisms (such as microRNAs) may also be involved.

MicroRNAs (miRNAs) are small, non-coding RNA molecules of 19–25 nucleotides that regulate gene expression post-transcriptionally through binding to the 3′-untranslated region (3′-UTR) of target mRNAs, causing mRNA cleavage, translational repression, or mRNA decay [3], [7] miRNAs have been found to regulate many critical cellular processes including apoptosis [8]–[11], differentiation [12]–[14], and cell proliferation [8], [13], [15], [16]. Aberrant expression of miRNAs is observed in most tumor types, indicating a role in carcinogenesis and a potential as prognostic/diagnostic biomarkers in cancer [17], [18]. We reason that miRNAs may be utilized as a better class of biomarkers due to their broad regulatory functions. In addition, the superior stability of miRNAs in formalin-fixed paraffin-embedded (FFPE) tissues and various body fluids (e.g., plasma, serum) further facilitates the clinical utility of miRNAs.

A number of recent studies have reported the expression profiles of miRNAs in endometrial cancer [19]–[26]. In this study, we aimed to investigate the prognostic value of miRNAs using archival FFPE samples of endometrial cancer patients. We systematically quantified the expressions of candidate miRNAs involved in critical cellular processes such as cell cycle control, chemoresistance, cell death and EMT transition (miR-26a, let-7g, miR-21, miR-181b, miR-192, miR-215, miR-200c, and miR-205) using quantitative real-time PCR (qRT-PCR) analysis. These miRNAs were chosen based on their critical target genes that are disrupted during endometrial carcinogenesis (e.g. p53, K-ras, PTEN) [27]–[32]. Our results show that the expression levels of miR-200c and miR-205 were significantly over-expressed in endometrial cancer. However, only the expression of miR-205 was significantly associated with patient survival. Previous studies have identified PTEN as one of the direct targets of miR-205 [33], [34]. Considering the significance of PTEN in endometrial cancer biology, we hypothesized that the miR-205 mediated PTEN inhibition mechanism might have an *in vivo* physiologic relevance in endometrial cancer. Indeed, our results have demonstrated that the expression levels of miR-205 were significantly inversely correlated with the expression levels of PTEN. As a result, our findings suggest that miR-205 may hold a unique potential as a biomarker for the prognosis of endometrial cancer.

## Results

### Expression of miRNAs in human endometrial cancer and evaluation of their prognostic values

**Figure 1:**
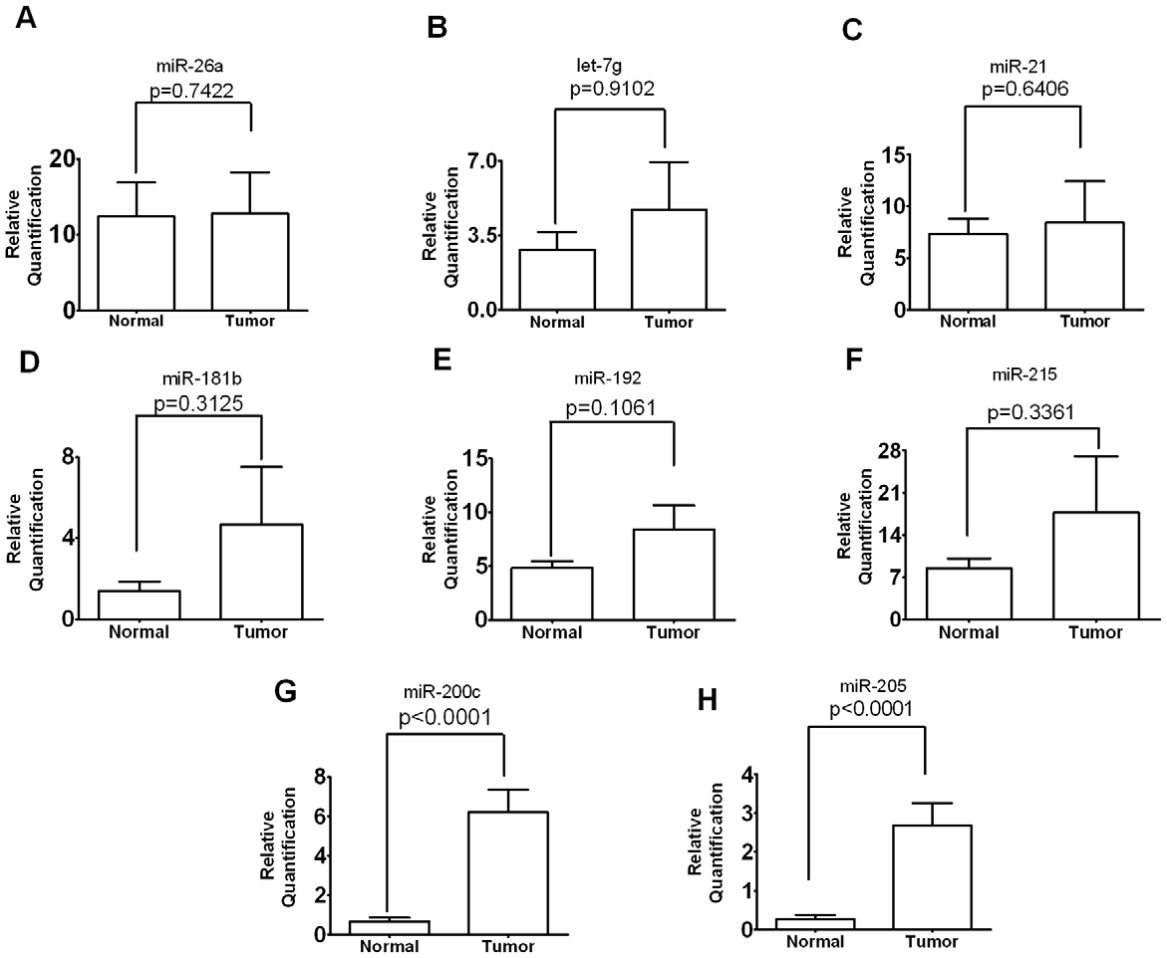
miRNA expression in normal and endometrial cancer tissue specimens. Relative quantification of miRNAs was expressed as normalized with an internal control RNU6B gene. (A) miR-26a expression (P = 0.742). (B) let-7g expression (p = 0.91). (C) miR-21 expression (P = 0.641). (D) miR-181b expression (P = 0.313). (E) miR-192 expression (P = 0.106). (F) miR-215 expression (P = 0.336). (G) miR-200c expression (P<0.0001). (H) miR-205 expression (P<0.0001). Statistical significance was calculated by a paired Student's t-test.

**Figure 2:**
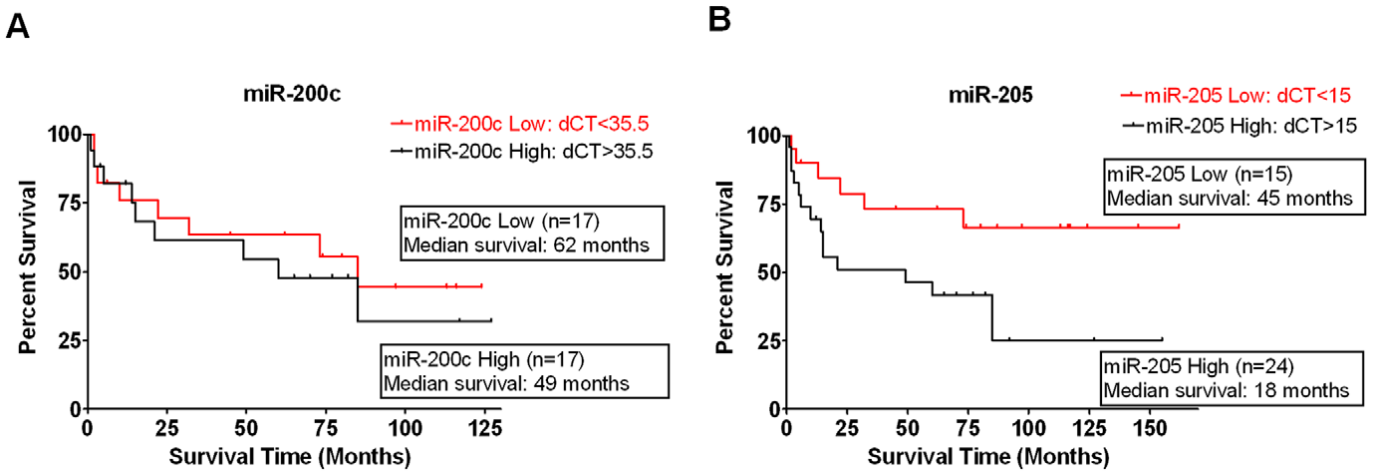
Relationship between miRNA expression and survival in endometrial cancer patients . Kaplan-Meier overall survival curves were plotted based on miRNA expression. (A) miR- 200c expression (P = 0.576, Logrank test). (B) miR-205 expression (P = 0.028, Logrank test).

The clinicopathologic parameters of the 48 endometrial cancer patients used in this study are described in Table 1. Half of the patients had endometrioid carcinoma, and the remaining patients were diagnosed with nonendometrioid histology (13 serous carcinoma, 5 clear cell carcinoma, 5 malignant mixed mullerian tumor (MMMT), and one undifferentiated carcinoma). Of these patients, 26 cases had stage I (54%), four cases had stage II (8%), 6 cases had stage III (13%), and 12 cases had stage IV (25%) of the disease. To investigate the clinical significance of the selected miRNAs in these endometrial cancer patients, we quantified their expression levels from 48 paired archival tumor and normal FFPE tissue specimens using real time qRT-PCR analysis. Of the miRNAs tested, there was no significant difference in the expression levels of miR-26a, let-7g, miR-21, miR-181b, miR-192, and miR-215 between tumor tissues and corresponding normal samples (P = 0.742; P = 0.91; P = 0.641; P = 0.313; P = 0.106; P = 0.336, respectively) (Figure 1A–F). In contrast, the expression of both miR-200c (P<0.0001; Figure 1G) and miR-205 (P<0.0001; Figure 1H) were significantly up-regulated in endometrial cancer specimens compared to adjacent normal tissues. To further analyze the significance of miR-200c and miR-205 in terms of clinical prognosis, Kaplan-Meier survival analysis was performed, using patient overall survival (Figure 2). Our results indicated that expression levels of miR-205 were significantly associated with patient survival. Patients with low levels (cut-off 15-fold of miR-205) tended to have better survival than patients with high levels of miR-205 (hazar ratio, 0.377; Logrank test, P = 0.028) (Figure 2B). This supports the notion that elevated levels of miR-205 in tumor tissues could lead to worse survival rates in endometrial cancer patients. However, expression levels of miR-200c were not associated with patient survival (P = 0.576; Figure 2A).

**Table 1:** Characteristics of endometrial cancer patients used for the study.

Clinicopathologic Parameters (n = 48)	n
**Pathology**	
Endometrioid carcinoma	24
Serous carcinoma	13
Clear cell carcinoma	5
Malignant mixed mullerian tumor (MMMT)	5
Undifferentiated carcinoma	1
**FIGO Stage**	
Endometrioid carcinoma	
Stage I	18
Stage II	2
Stage III	1
Stage IV	3
Serous carcinoma	
Stage I	4
Stage II	2
Stage III	2
Stage IV	5
Clear cell carcinoma	
Stage I	3
Stage IV	2
Malignant mixed mullerian tumor (MMMT)	
Stage I	1
Stage III	3
Stage IV	1
Undifferentiated carcinoma	
Stage IV	1

### Correlation of miR-205 and PTEN expression in endometrial cancer patients

**Figure 3:**
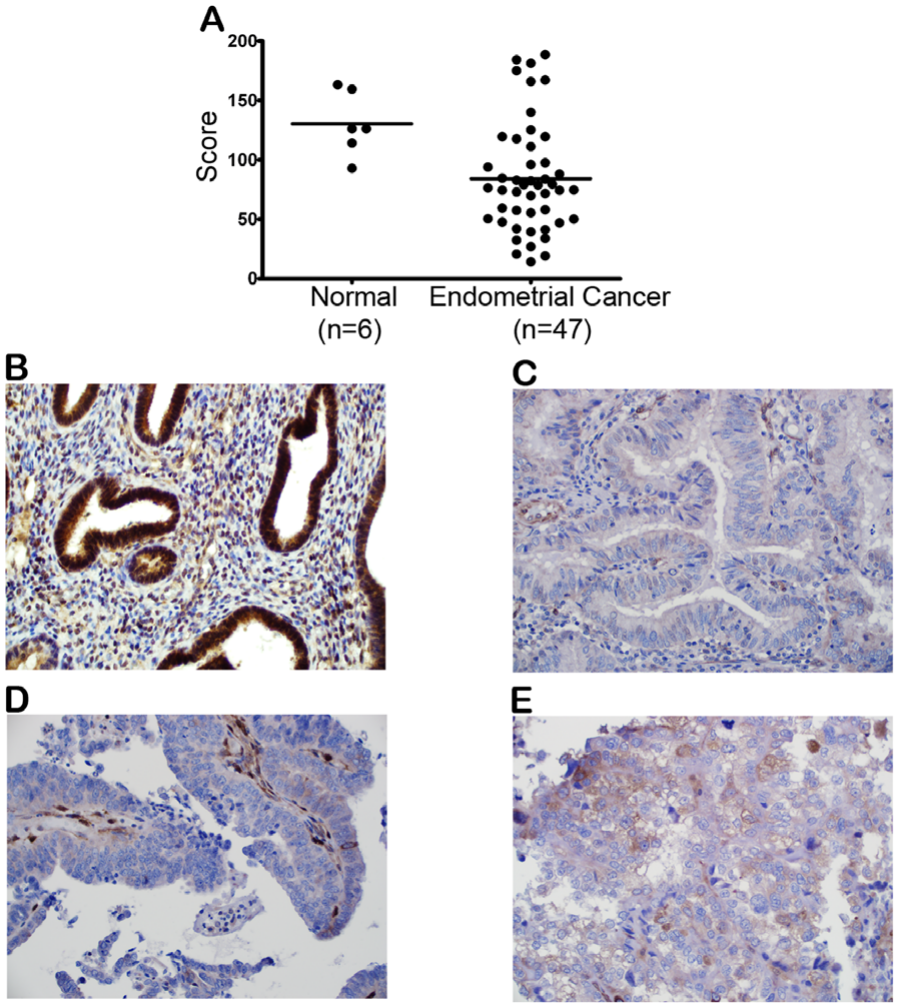
PTEN protein expression in endometrial cancer patients. (A) Scatter plot of immunostaining scores for PTEN in normal endometrium and endometrial cancer calculated by quantifying the staining intensity using ImageJ software. Bars represent the median for each category (normal median = 85, tumor median = 132). (B–E) PTEN protein expression is detected in normal endometrium (B), endometrioid carcinoma (C), serous carcinoma (D), and clear cell carcinoma (E) as detected by immunohistochemistry.

It has been reported that miR-205 targets a critical tumor suppressor gene PTEN [33], [34], which is frequently mutated or deleted in endometrial cancer [4]–[6]. However, genetic alterations cannot account for all PTEN protein loss observed in endometrial carcinomas, strongly suggesting the involvement of a post-transcriptional regulation in PTEN expression [35], [36]. Since the expression levels of miR-205 were elevated in tumor tissues, we reasoned that enhanced inhibition of PTEN by miR-205 might be a physiologically relevant mechanism during endometrial carcinogenesis. In order to investigate an *in vivo* relationship between miR-205 and PTEN protein expression, we constructed tissue microarrays (TMAs) from archival FFPE tissue specimens and performed immunohistochemistry with PTEN 6H2.1 antibody on sectioned TMAs. Our results indicate that PTEN protein expression was decreased in endometrial cancer compared to normal endometrium. Figure 3A shows that median expression of PTEN was significantly lower in tumor tissues (median score 85) compared to normal tissues (median score 132) (P = 0.0058). Figure 3 (B–E) shows examples of PTEN staining in normal endometrium (3B), endometrioid carcinoma (3C), serous carcinoma (3D), and clear cell carcinoma (3E).

**Figure 4:**
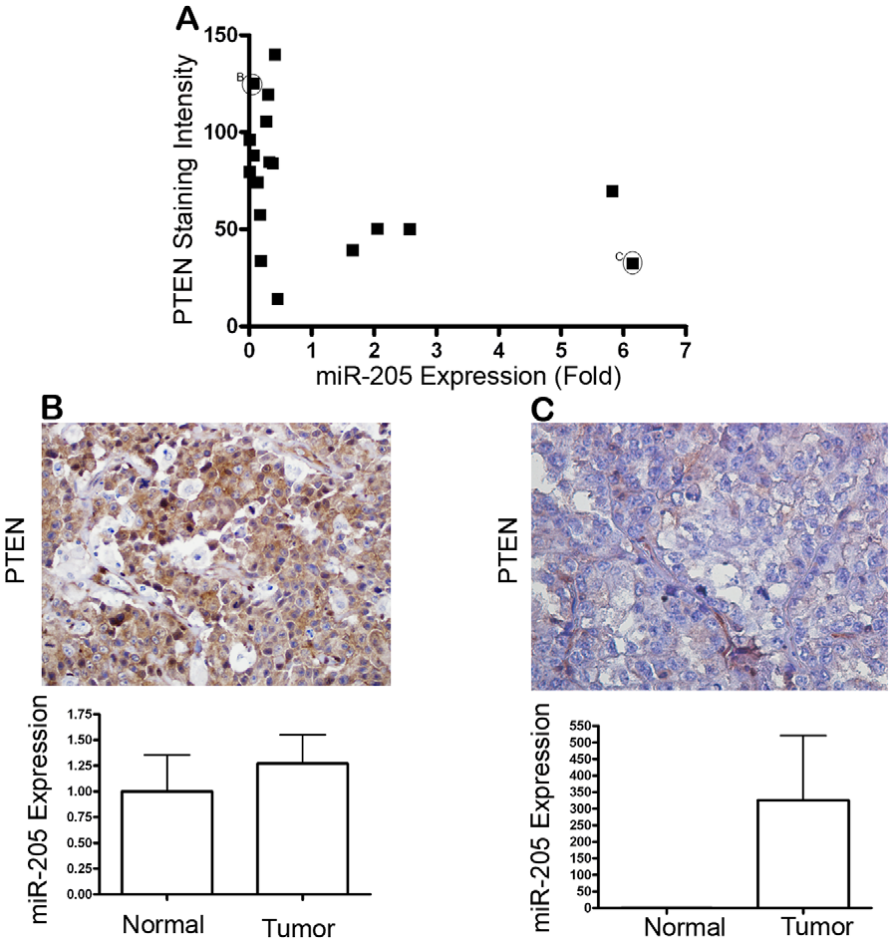
Correlation between miR-205 and PTEN expression in endometrial cancer patients. (A) Correlation of miR-205 and PTEN expression was analyzed by two-tailed Spearman nonparametric correlation test (P = 0.034, Spearman correlation coefficient = −0.502). PTEN protein expression accompanied by miR-205 expression (in triplicate) is illustrated in individual cases; undifferentiated carcinoma (B), and clear cell carcinoma (C).

To further confirm the inverse relationship between miR-205 and PTEN expression, we performed a non-parametric spearman correlation analysis by using the cases that have both miR-205 and PTEN protein quantification data. Our results indicate the presence of an inverse correlation in 69% of the cases (spearman correlation coefficient = −0.502, P = 0.034) (Figure 4A). Figure 4B and Figure 4C show examples of PTEN staining accompanied by miR-205 expression levels in individual cases. Figure 4B demonstrates an undifferentiated carcinoma with high PTEN staining and only a slight up-regulation in miR-205 expression between tumor and adjacent normal tissue (∼1.25 fold). Figure 4C demonstrates a clear cell carcinoma with very low PTEN staining and ∼350 fold up-regulation in miR-205 expression between tumor and adjacent normal tissue.

**Figure 5:**
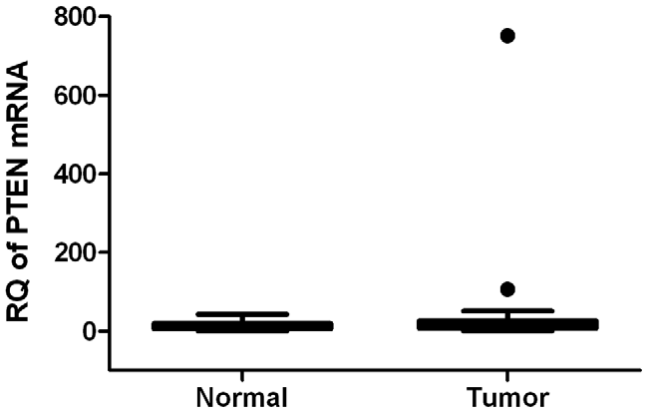
PTEN mRNA expression in endometrial cancer patients. Relative quantification of PTEN mRNA was expressed as normalized with an internal control β-actin gene (p = 0.31). Statistical significance was calculated by a paired Student's t-test.

Finally, to support our hypothesis that decreased PTEN protein expression is mediated by a post-transcriptional control, we quantified PTEN mRNA levels using real-time qRT-PCR. Our results indicate that PTEN mRNA expression levels in endometrial cancer specimens showed no difference when compared to adjacent normal tissues (Figure 5). These results suggest that the lack of correlation of PTEN mRNA with the loss of PTEN protein in endometrial cancer was likely due to a post-transcriptionally regulated mechanism.

## Discussion

In this study, we identified significantly over-expressed miR-200c and miR-205 in endometrial cancer compared to normal endometrial tissue (Figure 1). This finding is consistent with many other studies reporting elevated levels of these two miRNAs in different types of endometrial cancer [19]–[24], [26]. Therefore, we suggest that the over-expression signature of these miRNAs in endometrial cancer is not specific for histologic type. Chung *et al.* reported that the aberrant expression of miR-205 was correlated with advanced stage in endometrial cancer [22]. However, our results showed that miR-205 expression was not related to the stage disease or tumor type (Figure S1). We discovered that miR-205 expression was significantly associated with overall patient survival such that patients with higher levels of miR-205 tend to have a worse survival phenotype (Figure 2). Our results support a notion that miR-205 may have potential as a biomarker for the negative prognosis of endometrial cancer and therapeutic approaches targeting elevated levels of miR-205 should be explored as a novel approach to improve clinical outcomes.

The miR-200 family and miR-205 have also been reported to be dysregulated in other types of cancer. The miR-200 family has been reported to be up-regulated in ovarian cancer and cholangiocarcinoma [37]–[39], but down-regulated in hepatocellular carcinoma, breast cancer and renal cell carcinoma [40]–[42]. By contrast, miR-205 has been reported to be up-regulated in ovarian, bladder, and breast carcinomas [37], [43], [44], but down-regulated in prostate and esophageal cancers [45], [46]. These different expression patterns in some types of cancer may reflect the presence of tumor type-specific mechanisms that are mediated by regulatory miRNAs. The most well-known function of the miR-200 family and miR-205 is their ability to regulate the expression of E-cadherin transcriptional repressors ZEB1 (also known as δEF1) and SIP1 (also known as ZEB2), factors important for epithelial mesenchymal transition (EMT) and tumor metastasis [32]. However, miR-200 family and miR-205 function as tumor suppressors through the inhibition of EMT. Since the expression of these miRNAs is increased in the tumor samples, we reasoned that other proteins targeted by these miRNAs might be involved in endometrial cancer. One of the reported targets of miR-205 is a critical tumor suppressor gene, PTEN [33], [34].

PTEN expression has been reported to be reduced in endometrial carcinoma [4], thus it has a critical role in endometrial cancer biology [35], [36], [47], [48]. However, mutations/deletions of PTEN are not the only reason for the reduced PTEN expression. Rather, epigenetic mechanisms, such as promoter methylation, increased PTEN protein degradation or regulation through miRNAs are also important [49]. With this in mind, we analyzed the expression levels of PTEN mRNA and protein in endometrial cancer patients. We have shown that, although there was no change in the expression of PTEN mRNA, the expression of PTEN protein was decreased in tumor tissues compared to adjacent normal tissues. This supported the hypothesis that a post-transcriptional regulation of PTEN, possibly mediated by miRNAs, is valid. Together with the reports that PTEN is the direct target of miR-205, our results support the potential role of miR-205 in regulating PTEN in endometrial cancer.

In general, a decrease in PTEN expression is associated with endometriod carcinoma and may be a useful biomarker to distinguish endometrioid carcinoma from serous carcinoma [4]. However, other studies have reported no significant difference in PTEN expression, suggesting that PTEN may not always be useful to distinguish these tumor subtypes [50], [51]. Therefore, the clinical utility of PTEN as a biomarker remains to be determined. We reason that miR-205 may be a superior biomarker than PTEN due to its broad impact on multiple targets and pathways.

In summary, our results indicate that the expression of miR-205 and PTEN are inversely correlated in endometrial cancer patients, implying the physiologic significance of the miR-205 mediated PTEN inhibition mechanism in endometrial cancer biology. More importantly, the expression of miR-205 is associated with endometrial cancer patient survival. Thus, we reason that miR-205 may regulate PTEN and other protein expression in the pathogenesis of endometrial cancer. Future studies are clearly needed to fully validate the clinical utility of miR-205 as prognostic biomarker with multicenter large endometrial cancer patient cohorts.

## Materials and Methods

### Ethics

Clinical sample cohort used for this study was approved by the Institution Review Board of Stony Brook University Medical Center. Written informed consent was received from all participants involved in the study.

### Patients and Samples

Approval from the Institution Review Board was obtained for both the collection of tissues and the use of archival formalin-fixed paraffin embedded (FFPE) tissue blocks. For RNA extraction, tumor samples and the adjacent normal tissues were obtained from 48 endometrial cancer patients who underwent hysterectomy at Stony Brook University Hospital, Stony Brook, NY. The characteristics of these patients are shown in Table 1. For immunohistochemistry, tumor tissues were obtained from 47 endometrial cancer patients and non-pathologic endometrial tissues as normal controls were obtained from five non-endometrial cancer patients. Paraffin blocks containing formalin-fixed specimens of the tissues (FFPE) were acquired from the archival collections of the Department of Pathology and used for subsequent analyses. The specimens were selected from 1995 to 2010 with up to 15 years clinical follow up information.

### Tissue Microarrays (TMAs)

Tissue microarrays were prepared from FFPE blocks of hysterectomy specimens. Hematoxylin and Eosin stained sections from all cases were carefully reviewed. For each case, areas of endometrial cancer and normal endometrium were designated. Using the Advanced Tissue Arrayer (Model: ATA-100, Millipore, Billerica, MA, USA), with a 1.5 mm diameter needle, three cores were extracted from the area of tumor and three cores were removed from the benign endometrium for each case. The cores were embedded into a paraffin tissue microarray block in predetermined positions, with up to 60 cores in each tissue microarray and two additional cores placed to designate the orientation of the block.

### Immunohistochemistry (IHC) Analysis of PTEN Expression

TMAs were sectioned at 5 µm and the sections were heat immobilized on glass slides at 60°C overnight. After deparaffinization, antigen retrieval was performed in a citrate buffer [20 mmol/L (pH = 6)] at 120°C for 10 minutes followed by 3% hydrogen peroxide for five minutes. Staining was applied to FFPE tissues by using an avidin-biotin (ABC) method (Vector Laboratories, Burlingame, CA). Sections were incubated for one hour with a mouse monoclonal PTEN antibody, PTEN 6H2.1 (Cascade Biosciences, Winchester, MA) at a dilution of 1∶300. Monoclonal mouse IgG (BD Biosciences, Franklin Lakes, NJ, USA) was used as an isotype–negative control. PTEN staining was visualized using 3, 3′-diaminobenzidine (Dako, Carpinteria, CA), and sections were counterstained with dilute hematoxylin, dehydrated, and coverslipped for bright-field microscopy. Staining for IHC was quantified by averaging intensity from three cores of each case (ImageJ software (http://rsb.info.nih.gov/ij/)).

### RNA Isolation

Using archival FFPE tissues, separate areas of tumor and normal endometrium were identified using the corresponding Hematoxylin and Eosin stained sections and cores measuring 1.5 mm in diameter and 2 mm in length (approximately 0.005 g) were extracted. Subsequently, the samples were deparaffinized, hydrated, digested with proteinase K, and ultimately, total RNAs were isolated using TRIZOL reagent (Invitrogen, Carlsbad CA, USA).

### Real time qRT-PCR Analysis of miRNA Expression

The miR-26a, let-7g, miR-21, miR-181b, miR-192, miR-215, miR-200c, and miR-205 specific primers and the internal control RNU6B gene were purchased from Ambion (Applied Biosystems, CA, USA). cDNA synthesis was performed by the High Capacity cDNA Synthesis Kit (Applied Biosystems, CA, USA) with miRNA specific primers. Real-time quantitative RT-PCR (qRT-PCR) was carried out on an Applied Biosystems 7500 Real-time system (ABI 7500HT instrument) with miRNA specific primers by TaqMan Gene Expression Assay.

### Real time qRT-PCR Analysis of PTEN mRNA

In order to detect mRNA from FFPE tissues, cDNA synthesis was performed by the High Capacity cDNA Synthesis Kit (Applied Biosystems, CA, USA) with random primers. For qRT-PCR analysis, custom designed real time PCR primers and probes for PTEN and the internal control gene β-actin were used (Applied Biosystems CA, USA). The primers were designed to obtain as small amplicons as possible due to the concern about the degradation of mRNA in FFPE tissues [52]. The sequences of these primers/probes are listed in Table 2. qRT-PCR was carried out on an ABI 7500HT instrument with mRNA specific primers by TaqMan Gene Expression Assay under the following conditions: 50°C, 2 min of reverse transcription; 95°C, 10 min; 95°C, 15 s; 60°C, 1 min for up to 40 cycles (n = 3). Expression of PTEN mRNA was normalized according to the internal β-actin control, and the relative expression values were plotted.

**Table 2:** Sequences of PCR primers and probes used in qRT-PCR of PTEN mRNA.

Gene	Amplicon size(bp)	Forward Primer	Reverse Primer	Probe
PTEN	74	GTTCATAACGATGGCTGTGG	TGCATGCATGACACATTAACA	TTGCCACAAAGTGCCTCGTTTACC
β-actin	64	GCACCCAGCACAATGAAG	CGATCCACACGGAGTACTTG	CAAGATCATTGCTCCTCCTGAGCG

### Statistical Analysis

All statistical analyses were performed using GraphPad Prism software 5.0. Gene expression ΔCt values of miRNAs from each sample were calculated by normalizing according to internal control RNU6B expression, and relative quantification values were plotted. The differences between tumor and normal tissues were analyzed by a paired Student's t-test. Kaplan-Meier survival curves were generated to evaluate the expression levels of miR-200c and miR-205 with survival rate. Unpaired Student's t-test was used to analyze the difference in PTEN staining between tumor and normal tissues. Statistical significance was set up to P<0.05 in each test.

## Supporting Information

Figure S1 **Relationship between miR-205 expression and different stages of endometrial cancer.** miR-205 expression was expressed as normalized with an internal control RNU6B gene. (A) One-way ANOVA test was used to analyze the association of miR-205 expression in stage I, stage II, stage III and stage IV of endometrial cancer. (B) Unpaired Student's t-test was used to analyze the association of miR-205 expression in stage I&II *vs* stage III&IV.
(TIF)
