# Supplementary figures and images for: Prognostic Significance of miR-205 in Endometrial Cancer
Source: PLoS One. 2012 Apr 13;7(4):e35158. doi: 10.1371/journal.pone.0035158 (PMC3325973; doi:10.1371/journal.pone.0035158)

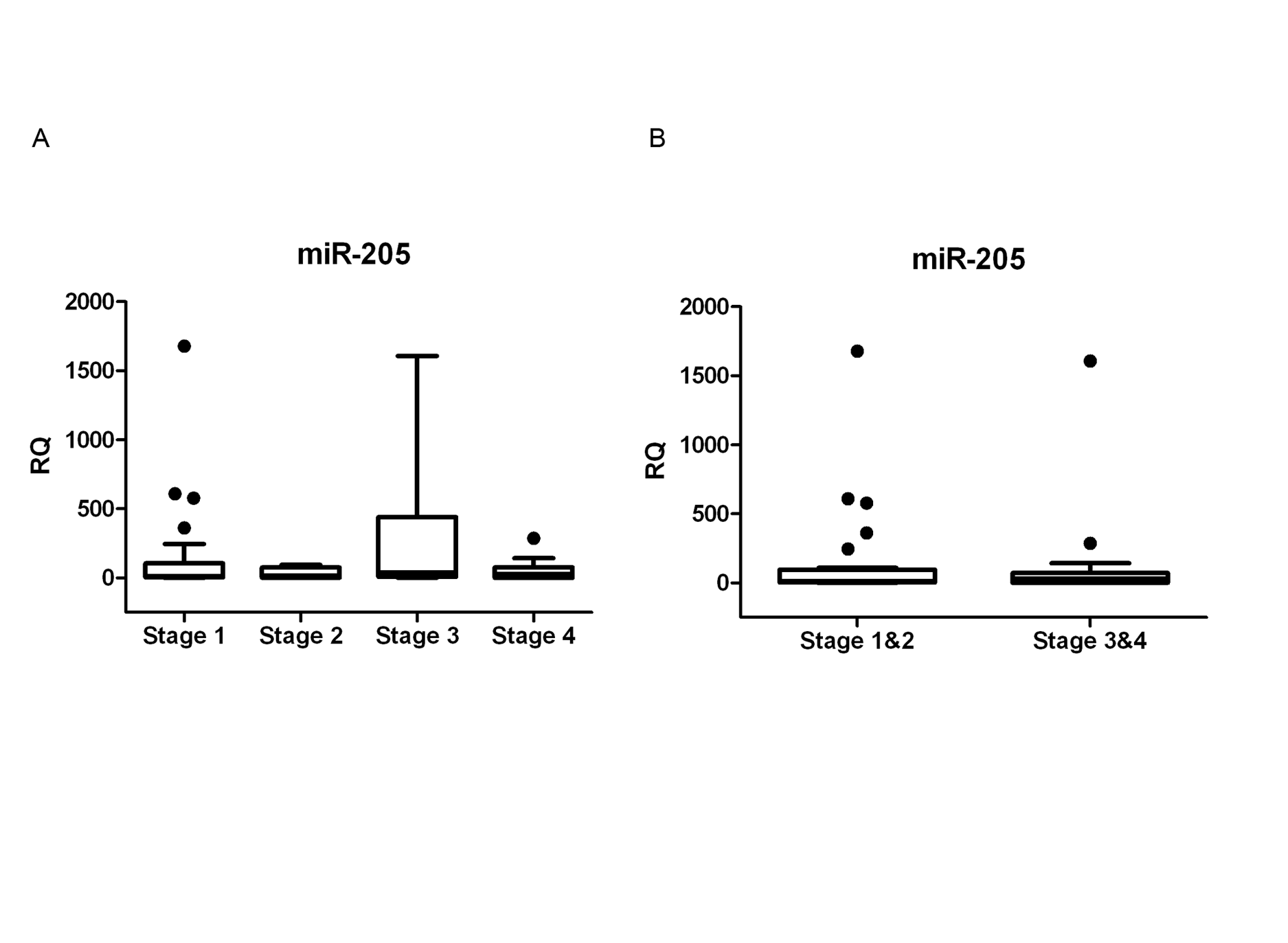

Supplement: Figure S1 — Relationship between miR-205 expression and different stages of endometrial cancer. miR-205 expression was expressed as normalized with an internal control RNU6B gene. (A) One-way ANOVA test was used to analyze the association of miR-205 expression in stage I, stage II, stage III and stage IV of endometrial cancer. (B) Unpaired Student's t-test was used to analyze the association of miR-205 expression in stage I&II vs stage III&IV. (TIF) [file pone.0035158.s001.tif]
